# SARS-CoV-2 and the role of fomite transmission: a systematic review

**DOI:** 10.12688/f1000research.51590.1

**Published:** 2021-03-24

**Authors:** Igho J. Onakpoya, Carl J. Heneghan, Elizabeth A. Spencer, Jon Brassey, Annette Plüddemann, David H. Evans, John M. Conly, Tom Jefferson

**Affiliations:** 1 Nuffield Department of Primary Care Health Sciences, University of Oxford, Oxford, OX2 6GG, UK; 2 Trip Database Ltd, Newport, NP20 3PS, UK; 3 Department of Medical Microbiology & Immunology, Li Ka Shing Institute of Virology, Alberta, Canada; 4 University of Calgary and Alberta Health Services, Calgary, AB T2N 4Z6, Canada

**Keywords:** Fomites, transmission, COVID-19, systematic review

## Abstract

**Background:** SARS-CoV-2 has been detected in fomites which suggests the virus could be transmitted via inanimate objects. However, there is uncertainty about the mechanistic pathway for such transmissions. Our objective was to identify, appraise and summarise the evidence from primary studies and systematic reviews assessing the role of fomites in transmission.

**Methods: **This review is part of an Open Evidence Review on Transmission Dynamics of SARS-CoV-2. We conduct ongoing searches using WHO Covid-19 Database, LitCovid, medRxiv, and Google Scholar; assess study quality based on five criteria and report important findings on an ongoing basis.

**Results:** We found 64 studies: 63 primary studies and one systematic review (n=35). The settings for primary studies were predominantly in hospitals (69.8%) including general wards, ICU and SARS-CoV-2 isolation wards. There were variations in the study designs including timing of sample collection, hygiene procedures, ventilation settings and cycle threshold. The overall quality of reporting was low to moderate. The frequency of positive SARS-CoV-2 tests across 51 studies (using RT-PCR) ranged from 0.5% to 75%. Cycle threshold values ranged from 20.8 to 44.1. Viral concentrations were reported in 17 studies; however, discrepancies in the methods for estimation prevented comparison. Eleven studies (17.5%) attempted viral culture, but none found a cytopathic effect. Results of the systematic review showed that healthcare settings were most frequently tested (25/35, 71.4%), but laboratories reported the highest frequency of contaminated surfaces (20.5%, 17/83).

**Conclusions:** The majority of studies report identification of SARS-CoV-2 RNA on inanimate surfaces; however, there is a lack of evidence demonstrating the recovery of viable virus. Lack of positive viral cultures and variation in cycle thresholds create uncertainty about fomites as a mode of transmission. Heterogeneity in study designs and methodology prevents comparisons of findings across studies. Standardized guidelines for conducting and reporting research on fomite transmission is warranted.

## Introduction

The SARS-CoV-2 (COVID-19) pandemic is a major public health concern. According to WHO statistics, there have been over 90 million confirmed cases and over two million deaths globally as of 18th January 2021 ^1^. Although many national governments have implemented control measures and vaccines are now being approved and administered, the rate of infection has not subsided as anticipated. Understanding the modes of transmission of SARS-CoV-2 is critical to developing effective public health and infection prevention measures to interrupt the chains of transmission ^2^. Current evidence suggests SARS-CoV-2 is primarily transmitted via respiratory droplets and direct contact ^3^, but other transmission routes have been suggested – aerosol and fomites. 

While the respiratory, airborne, and direct contact modes of transmission have been investigated in detail, the role of fomites in the transmission of SARS-CoV-2 is less clear. Findings from previous systematic reviews have shown that viruses from the respiratory tract, such as coronaviridae, can persist on inanimate surfaces for some days ^4^, and it has been reported that SARS-CoV-2 can be transmitted indirectly through fomites or surfaces ^5^. However, some authors have suggested that there is a low risk of transmission of SARS-CoV-2 through fomites ^6, 7^ and others have suggested that the risk of such transmission is exaggerated ^8^.

Several studies investigating the role of fomites in SARS-CoV-2 are now being published but the evidence from such studies has not been systematically evaluated. The objective of this review was to identify, appraise and summarize the evidence from primary studies and systematic reviews investigating the role of fomites in the transmission of SARS-CoV-2. Terminology for this article can be found in Box 1.

**Box 1. Terminology**

**Table:** 

**Fomite:** Object or surface contaminated by infected droplets. The contamination can occur through sneezing, coughing on, or touching surfaces ^1^
**Viral load:** A measure of the number of viral particles present in an individual ^2^
**Cycle threshold:** The number of cycles required for the fluorescent signal to cross the threshold. Ct levels are inversely proportional to the amount of target nucleic acid in the sample ^3^

^1^World Health Organization. Q&A: How is COVID-19 transmitted? https://www.who.int/vietnam/news/detail/14-07-2020-q-a-how-is-covid-19-transmitted ^2^ https://www.cebm.net/covid-19/sars-cov-2-viral-load-and-the-severity-of-covid-19/ ^3^ https://www.ncbi.nlm.nih.gov/pmc/articles/PMC7521909/

## Methods

We are undertaking an open evidence review investigating factors and circumstances that impact on the transmission of SARS-CoV-2, based on our published protocol last updated on the 1 December 2020 (archived protocol: *Extended* data: Appendix 1 ^9^; original protocol: https://www.cebm.net/evidence-synthesis/transmission-dynamics-of-covid-19/). Briefly, this review aims to identify, appraise, and summarize the evidence (from studies peer-reviewed or awaiting peer review) relating to the role of fomites in the transmission of SARS-CoV-2 and the factors influencing transmissibility. We conducted an ongoing search in WHO Covid-19 Database, LitCovid, medRxiv, and Google Scholar for SARS-CoV-2 for keywords and associated synonyms. The searches for this update were conducted up to 20th December 2020. No language restrictions were imposed (see *Extended data:* Appendix 2 for the search strategies ^9^).

We included studies of any design that investigated fomite transmission. Predictive or modelling studies were excluded. Results were reviewed for relevance and for articles that looked particularly relevant, forward citation matching was undertaken and relevant results were identified. We assessed the reporting quality of included studies using five domains from the QUADAS-2 criteria ^10^; we adapted this tool because the included studies were not designed as diagnostic accuracy studies. We extracted the following information from included studies: study characteristics, population, main methods, and associated outcomes including the number of swab samples taken with frequency and timing of samples, and cycle thresholds and samples concentrations where reported. We also extracted information on viral cultures including the methods. One reviewer (IJO) assessed the risk of bias and extracted data from the included studies, and these were independently checked by a second reviewer (EAS). We presented the results in tabular format, and bar charts used to present the frequency of positive tests. Because of substantial heterogeneity across the included studies, we did not perform a meta-analysis.

## Results

**Figure 1.**
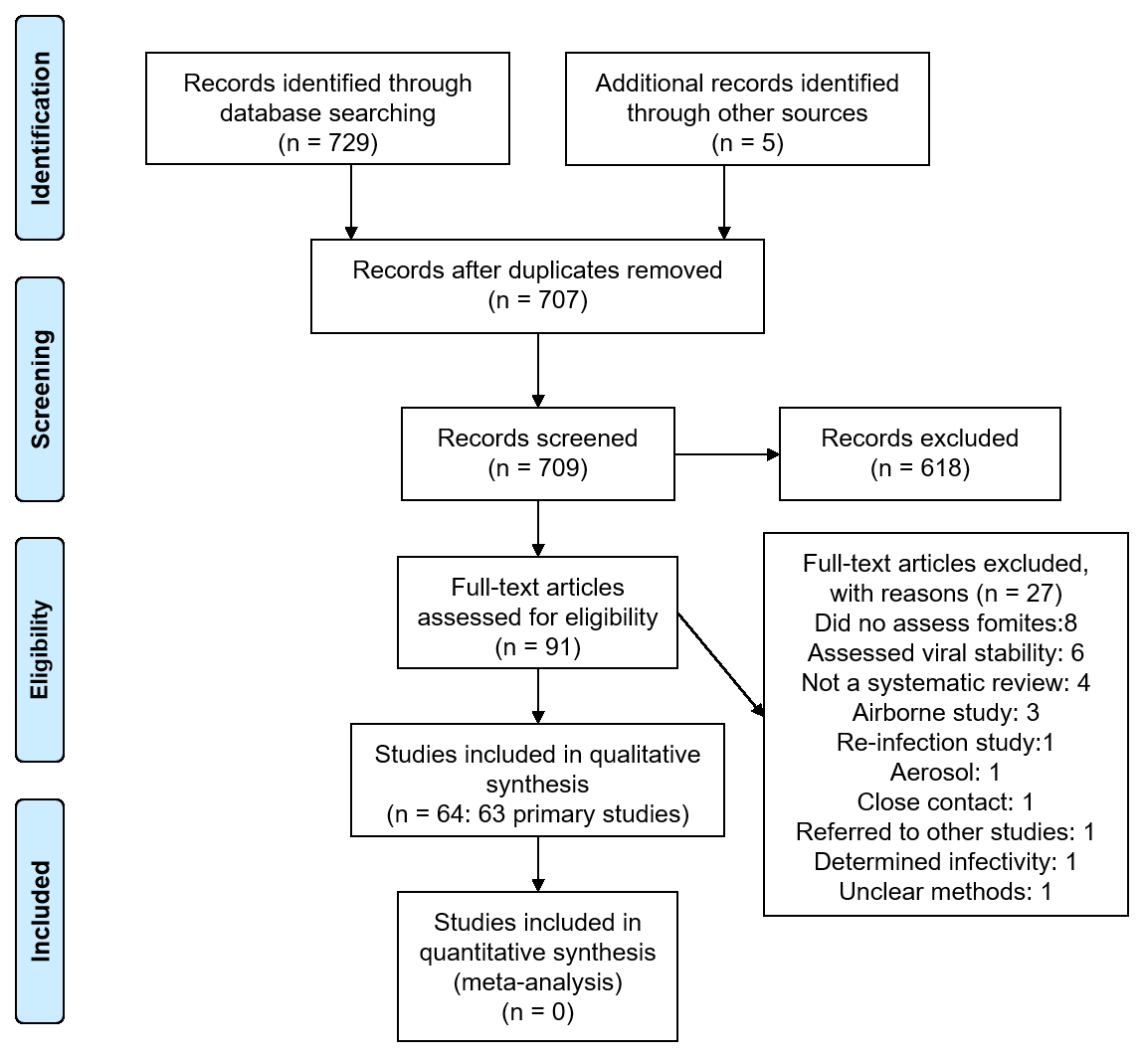
Flow diagram showing the process for inclusion of studies assessing fomites transmission in SARS-CoV-2.

We identified 709 non-duplicate citations of which 91 were considered eligible ( Figure 1). We excluded 27 full-text studies because they did not meet our inclusion criteria (see *Extended data:* Appendix 3 ^9^ for the list of excluded studies and reasons for exclusion). Finally, we included 64 studies: 63 primary studies and one systematic review (see *Extended data:* Appendix 4; characteristics of studies in Table 1 and Table 2 ^9^).

**Table 1.** Primary studies characteristics.

Study ID (n=63)	Setting	Sources of fomites	Number of swab samples taken	Viral culture	Notes
Abrahão 2020	Public places in urban area Brazil April 2020	15 bus stations, front-door sidewalk of 8 hospitals, 4 bus terminals, 3 benches and tables in public squares	101	No	Densely populated area. Ct<40 considered positive
Akter 2020	2 southern districts of Bangladesh over a 3- month period	Banknotes in circulation 6 non-issuable banknotes spiked with SARS-CoV-2 positive nasopharyngeal samples	850: both sides of each banknote from circulation swabbed	No	Circulating banknotes of varying denominations were collected from retail shops, ticket vendors and auto rickshaw drivers. intercity transport authority were regulated to ensure wearing masks, maintain social distancing (carrying 50% of total capacity) with personal hygiene.
Amoah 2020	2 peri-urban informal settlements in South Africa September 2020	Cistern handle, toilet seat, floor surface in front of the toilet, internal pull latch of cubicle door and tap in wash hand basin	68	No	Sampling was done twice in September 2020 when the reported active clinical cases were low in South Africa.
Ben-Shmuel 2020	COVID-19 isolation units in two hospitals and one quarantine facility in Israel	***Mild COVID-19:*** Floor, bed rails, bedside table, faucet handle, mobile phones, eyeglasses, patient's walker, air sampling filter ***Severe COVID-19:*** Bed rails, faucet handle, ventilator, staff computer mouse, staff mobile phone, bedside table, trash bin top, bench top, air sampling filter ***Patient's toilets:*** Toilet seat, handle grip, door handle ***Nurse station:*** Floor, bench top, computer mouse, staff mobile phone, glucometer, electric thermometer, BP cuff, air sampling filter ***Doffing area:*** Floor, door handle, trash bin top, air sampling filter	Smaller objects were swabbed entirely: 2 wet swabs plus 1 dry swab. **COVID-19 isolation units of** **Hospitals** ***Patient rooms*** (1–3 patients in mild condition): 21 samples ***Ventilated patients' rooms*** (invasive and non-invasive: 13 samples ***Patient's toilets:*** 4 samples ***Nurse station:*** 8 samples ***Doffing area:*** 4 samples **Quarantine hotel for ** **asymptomatic and mild** ** COVID-19 patients** Hotel room: 21 samples Public spaces: 21 samples	Yes	Patients stayed in private rooms either alone or as a family, but were free to move around the hotel and socialize in public spaces. **Viral culture method:** Vero E6 cells. CPE observed after 5 days.
Bloise 2020	Laboratory Spain	High-touch surfaces: Landline, barcode scanner, mobile phone, mouse, keyboard, environmental	22	No	
Cheng 2020	Environmental surveillance in hospital, Hong Kong	Bench, bedside rail, locker, bed table, alcohol dispenser, and window bench	Not reported	No	Close contact referred to those with unprotected exposure, defined as HCWs who had provided care for a case patient with inappropriate PPE and patients who had stayed within the same cubicle of the index case regardless of the duration of exposure.
Cheng 2020a	Hospital AIIRs in China February-March 2020	Bed rail, locker, bed table, toilet door handle, and the patient’s mobile phone	377	No	21 patients. 12 air changes per hour. Samples were collected before daily environmental disinfection.
Chia 2020	Hospital rooms of infected patients Singapore	Floor, bedrail, locker handle, cardiac table, electric switch, chair, toilet seat and flush, air exhaust vent	245	No	12 air changes per hour.
Colaneri 2020	Referral hospital in Northern Italy 21 to 29 February 2020	Buffer zone of patients' rooms: Door handles, waste container covers, sink handles, wall surfaces Doctors' and nurses' lounge: Kitchen table and sink, desks, computer keyboards, medical charts and parameters, tabs, door handles, therapy trolleys Staff personal belongings: Mobile phones	16	No	HCWs involved in the direct care of patients used PPE. Standard cleaning procedures were in place.
Colaneri 2020a	Infectious Disease Emergency Unit of a hospital in Italy	Rooms of patients with CPAP helmet, room of patient in high-flow oxygen therapy, PPE, staff equipment	26	Yes	HCWs involved in the direct care of patients used PPE. Standard cleaning procedures were in place. Air change in our wards is typically 7 volumes per hour. Swabs were performed around 12 noon, approximately 4 hours after cleaning. **Viral culture method:** Vero E6 cell line. CPE observed at 7 days
D'Accolti 2020	Acute COVID-19 ward of an Italian hospital	Inside: Floor, bedside table, bathroom sink, and bed headboard Outside: Ward corridor, nurse area and door, and warehouse shelves	22	No	Standard cleaning procedures twice daily in the morning and afternoon. Sampling was performed seven hours after cleaning. All staff wore PPE.
Declementi 2020	COVID-19 non-Intensive Care Unit Italy May 2020	Bed rail, sheets and pillow, floor and wall within 1m of bed, surgical mask, disposable gowns	24	No	Sampling: 1st day - 18 hrs after disinfection; 2nd day - 24 hrs. 12 samples were collected before extra- ordinary sanitization procedures and 12 after extra-ordinary sanitization procedures.
Ding 2020	The Second Hospital of Nanjing, China February 2020	Four isolation rooms, a nursing station, a corridor, an air-conditioning system, and other spaces in the airborne infectious- disease	107	No	10 patients. A sample was defined as positive at a Ct ≤38, and weakly positive at a Ct of 37–38. All HCWs used PPE. Sampling done before disinfection. Cleaning and disinfection of these rooms was conducted twice daily.
Döhla 2020	High-prevalence community setting with Germany's first largest high-prevalence cluster with regard to COVID-19 known at that point of time in March 2020	Electronic devices, Knobs and handles, Plants and animals, Furniture, Food and drinks, Clothing	119	Yes	Quarantined households. No standardised environmental sampling was carried out. No characterization of cleaning methods or materials was performed. **Viral culture method:** Vero E6 cells. CPE observed after "several days".
Escudero 2020	Multipurpose ICU and a cardiac ICU in Spain All patients had high level of disease severity 16 to 27 April 2020	Door knob, chair telephone, computer keyboard, computer mouse, sink faucet, perfusion pump, cart, door handle, ICU workers’ shoe sole, table bench, bed, bed rail, mattress, ventilator, bag valve mask, BP cuff, ECG electrodes, oxygen supply system, sling, waste container, tracheal tube	102	No	All the ICU units were equipped with negative pressure of –10 Pa and an air flow circuit with circulation from the central area to the boxes with an air change rate of 20 cycles/hour. All staff used PPE. Standard cleaning was performed twice daily (morning and afternoon).
Feng 2020	Frequently touched surfaces in hospital isolation wards China 13/02/2020 to 05/03/2020	***Public surfaces in the isolation room:*** Door handles, window handle, lavatory door handle, lavatory floor, lavatory floor drain, toilet seat, toilet flush button, and faucet ***Private surfaces in the isolation room:*** Patient’s toothbrush, mouthwash cup, towel, pillow, bed sheet, bedrails, bedside table, bedside wall above the patient’s head, bedside floor, kettle handle, and cup	202 Private surfaces: 132 Public surfaces: 70	Yes	**Viral culture method:** Not specified
Fernández- de-Mera 2020	Isolated rural community in Spain with a high COVID‐19 prevalence 13/05/2020 and 05/06/2020	***Households:*** Toothpaste tubes, fridge and oven handles, and the main door handle ***Public service areas:*** Keyboards, tables, chairs, refrigerators and entry door handles	55	No	
Ge 2020	Hospital wards in 3 different hospitals (ICU plus hospital ward) February 2020	Door handle, computer keyboard, nurses' station , urinal, bedhead, passage way, weighing scale, handrail, medical record rack	112	No	The 3 hospitals with different protection levels. Ct value <40 was considered as positive. Routine disinfection was acted every 4 h in ICU. Samples collected 1–3 times in surfaces across sites
Guo 2020	Hospital Wards, Wuhan, China February 19 through March 2, 2020	Floors, computer mice, trash cans, sickbed handrails, patient masks, personal protective equipment, and air outlets	105	No	
Harvey 2020	Public locations and essential businesses Massachusetts, USA March-June 2020	High-touch nonporous surfaces likely to be contaminated with SARS-CoV-2 during an outbreak: a trash can, liquor store, bank, metro entrance, grocery store, gas station, laundromat, restaurant, convenience store, post office box, and crosswalks.	348	No	Observed a total of 1815 people and 781 bare-hand touches across all sites from April 23 to June 23. Mean temperature on sampling days was 17°C, the mean relative humidity 61%
Hu 2020	Hospitals with COVID-19 patients in Wuhan, China 16 February to 14 March, 2020	Cabinet, patient's bedrail, door handle and patient monitor	24	No	
Hu 2020a	Quarantine room, Qingdao, China Before and after study March 2020	Corridor, bathroom, bedroom, living room - high-frequency touch surfaces	46	No	All sites were sampled 3 times - 1st sample 4 h after case confirmation; subsequent samples were taken within 24 h after every disinfection. A Ct value <37 was defined as positive, Ct value of ≥40 was defined as a negative.
Jerry 2020	ED, ICU, HDU, 6 medical wards Dublin, Ireland 5th May and 15th May 2020	Patient room housing a laboratory- confirmed COVID-19 patient; empty patient room following terminal cleaning and UVC decontamination carried out after the discharge of a laboratory-confirmed COVID-19 case; and the nurses' station of each of the wards with COVID-19 patients.	81	No	Timing of surface swab samples was determined by passage of time from most recent clean. COVID-19 patient rooms were cleaned once daily and nurses' station areas twice. For swabs of these areas, a minimum time of 4 h was allowed to elapse before samples were taken
Jiang 2020	2 isolation areas at the First Hospital of Jilin University, China	Door handle, general surface, consulting rooms, observation rooms, laboratory, buffer room, keyboard, thermometers, window frames, PPE	130	No	15 patients.
Jiang 2020a	2 rooms of a quarantine hotel China March 2020	Door handle, light switch, faucet, bathroom door handle, toilet seat, flush handle, thermometer, TV remote, pillow cover, duvet cover, sheet, towel	22	No	2 patients. Ct <40 was considered positive.
Jin 2020	ICU in hospital, China March 11, 2020	Armrests on the patient’s bed, desk surface of patient’s ward area, door handles, desk surface of the nurse's station, computer keyboard at the nurse's station	5	No	The ICU was routinely cleaned three times daily. All staff wore PPE. Sampling done 2 hours after the completion of routine cleaning
Kim 2020	Hospitalised patients with COVID-19, South Korea March 25 to April 8, 2020	Bed rails, medical carts, the floor, door handles, the bathroom sink, the toilet, and other fomites (e.g., cell phones, intercoms, and TV remote controllers)	220	No	Medical staff used PPE and everyone in the hospital was encouraged to wear masks and follow hand hygiene practices.
Lee 2020	6 hospitals and 2 mass facilities in South Korea February-March 2020	Frequently touched surfaces in wards (telephones, bedrails, chairs, and door handle) and communal facilities of COVID- 19 patients in the hospital.	80	No	Disinfection and cleaning had been performed by the local health centers before samples were collected from hospitals. No prior disinfection and cleaning procedures in mass facilities. Ct<35 was considered positive
Lei 2020	ICU and an isolation ward for COVID‐19 patients, China	***Patient areas:*** Floor, bedrail, bedside table, patient clothing, bedsheet, control panel of ventilator, ventilator outdoor valve, mobile phone, toilet, bathroom door handle, sink faucet handles ***Healthcare workers area:*** Changing room door handle, floor, sink faucets, keyboard mouse of mobile computer, handle of mop used by the cleaning staff	182	No	Two samples collected in the morning. Average air changes per hour were 240–360. The floor of the ICU was cleaned twice a day, at 11 am and 3 pm The furniture and equipment in the ward are also cleaned once a day at 11 am. CT<40 was considered positive.
Lui 2020	Hospital in Hong Kong	Disposable chopsticks	14	No	5 consecutive asymptomatic and postsymptomatic patients.
Lv 2020	Laboratory, China, Feb and Mar 2020	Door handle, elevator buttons, handles of sample transport boxes, surfaces of lab testing equipments, PPE, lab floor	61	No	
Ma 2020	COVID-19 patients in ICU and hospital wards in China	***COVID-19 patients:*** Toilet seat and handle, patient transport cart, floor, pillowcase, corridor handrail, seat pedal, hands, ventilation duct, computer keyboard, faucet handle, toilet flush button, remote control, table top, door handle ***Control group:*** Table top, pillow towel, mobile phone, toilet pit, toilet exhaust fan	242	No	
Maestre 2020	2 home-quarantined subjects in the USA	Floors, toilet door handle, AC filter, sink handle, toilet seat, door knob, refrigerator handle, high chair, phone screen, couch TV top surface, dining table	22	No	Home was naturally ventilated one hour per day, in the early morning; HVAC temperature setting was kept at 23.9°C day and night with the air conditioner; average relative humidity 56.6%. 1 home was cleaned daily; other home was cleaned 2–3 times/week. Samples collected 2 months after onset of symptoms (one month after COVID-19 symptoms had resolved in the household)
Marshall 2020	9 workplace locations in Europe and the USA	24 high-frequency-touch point surfaces: Office desks, door handles, entrance push button, faucet handles, log book, control panels, file drawer handle, mouse, keyboard, elevator button, refrigerator handle, work bench, plastic bin	Locations with positive employees: 2400 Locations without positive employees: 3000	No	Sampling occurred near the end of work shifts and before surfaces were cleaned and disinfected. Five surfaces were swabbed daily during the study and were considered the 5 greatest-risk sentinel surfaces. Ten surfaces were swabbed daily and were rotated among the remaining locations and were considered systematic surfaces. Ct≤38 was considered positive. Both RT-PCR and serology.
Moore 2020	Hospitalised patient in the UK 3rd March 2020 to 12th May 2020	Toilet door handle, door handle, nurse call button, portable vital signs monitor, bed rail, bed control, monitor, syringe driver, bedside computer, chair arm, curtain, window sill, air vent, trolley drawer	336	Yes	11 negative pressure isolation rooms. **Viral culture method**: Vero E6 cells. CPE observed at 7 days.
Nakamura 2020	Hospitalised COVID- positive patients in Japan January 29th to February 29th, 2020	Ventilation exits, phones, tablets, masks, PPE, stethoscopes, blood pressure cuffs, intubation tubes, infusion pump, pillows, TV remote controls, bed remote controls, syringes, patient clothes, personal data assistants, personal computers, computer mouse, consent form paper, patient palm, pulse oximeter probe, door knobs, bed guardrails, over tables, touch screen of ventilator, monitor, nurse call buttons, TV, curtains, toilet seats, hand soap dispensers, window sill, exhaust port, door sensor	141	No	Environmental samples from all rooms (except Room 2) were collected after 6–8 hours of daily room cleaning and disinfection. Room 2 was cleaned and items were disinfected at least once a day.
Nelson 2020	Long-term care facilities undergoing COVID-19 outbreaks, Canada	High-touch surfaces, communal sites, and mobile medical equipment	89	No	
Ong 2020	ICU ward of hospital in Singapore	Bedrail, floor, stethoscope, surgical pendant, ventilators, air outlet vents, infusion pumps, glass window, cardiac table	200	Yes	Routine twice-daily environmental cleaning. All sampling was conducted in the morning before the scheduled environmental cleaning (ie, the last cleaning time was the afternoon prior to environmental sampling). **Viral culture method:** Vero C1008 cells. CPE observed at 7 days.
Ong 2020a	Dedicated SARS-CoV-2 outbreak center (isolation rooms) in Singapore Jan-Feb 2020	Infection isolation rooms (12 air exchanges per hour) with anterooms and bathrooms, PPE	38	No	One patient’s room was sampled before routine cleaning and 2 patients’ rooms after routine cleaning. Twice- daily cleaning of high-touch areas was done using 5000 ppm of sodium dichloroisocyanurate. The floor was cleaned daily.
Ong 2020b	HCWs caring for confirmed COVID-19 patients in a hospital in Singapore	PPE	90	No	15 patients. The median time spent by HCWs in the patient’s room overall was 6 minutes (IQR, 5–10). Activities ranged from casual contact (eg, administering medications or cleaning) to closer contact (eg, physical examination or collection of respiratory samples).Gloves and gowns were not swabbed because these are disposed after each use.
Pasquarella 2020	Single hospital room with elderly COVID-19 patient Italy	Right bed rail, the call button, the bed trapeze bar, the stethoscope; moreover, the patient’s inner surgical mask	15	No	Surfaces sampling was carried out two days after the patient’s second positive swab (Ct 24), 7 days after hospitalization. The surfaces sampling was carried out 2 hours after cleaning and disinfection procedures.
Peyrony 2020	ED at a university hospital, France April 1 to April 8, 2020	**Patient care area:** Stretchers, cuffs for arterial blood pressure measurement, pulse oximeter clips, stethoscopes, ECG or ultrasound (US) devices, trolleys, monitor screens, benches, inside door handle, oxygen delivery manometer, plastic screen between patients, and floor. **Non-patient care area:** Patients waiting room, corridor with personal protective equipment (PPE) storage, staff working rooms, refreshment room, toilets, changing room, research office and medical equipment stockroom	192	Yes	Air exchange rate in the different rooms where the samples were made ranged from 1 to 7 m3/h and room sizes from 30 to 60 m3, thus the entire air renewal duration of these rooms could range from 4 to more than 24 h. Monitoring room and staff working rooms were regularly decontaminated every 2 or 3 h. HCWs wore PPEs. **Viral culture method:** Not specified
Piana 2020	Hospital in Italy May-June 2020	Indoor surfaces from three COVID- reference hospitals, buildings open to public use (1 office, 1 fast food, 1 church), outdoor areas, used handkerchiefs with nasopharyngeal secretions.	92	No	CT values ≤40 were considered positive.
Razzini 2020	COVID-19 ward of hospital in Italy May 12, 2020	Corridor for patients, ICU, undressing room, locker/passage for medical staff, dressing room	37	No	Negative airflow system. Sampling was carried out before daily cleaning operations. Temperature was 20° to 22 °C and relative humidity 40 to 60%. Medical and paramedical staff used PPE. Ct value was ≤40 was considered positive.
Ryu 2020	2 different hospital settings in South Korea March 2020	Patient monitor, ventilator monitor, HFNC, blood pressure cuff, pillow, suction bottle and line, Ambu bag, infusion pump, wall oxygen supply, fluid stand, door button or knob, bed side rail, head and foot of the bed, nurse call controller, lower part of the window frame, top of the television [TV], air exhaust damper, wall and floor of the room, toilet paper holder, and inside and seat of the toilet); the anteroom (ie, door button, keyboard, mouse, and floor); the floor of an adjacent common corridor; and the nursing station (ie, counter, interphone, keyboard, mouse, chair, and floor).	No	No	Negative pressure rooms (A); 2 common 4-bed rooms without negative pressure and ventilation systems (B). Room cleaning, and disinfection were not performed every day due to the shortage of PPE and vague fears of cleaners.
Santarpia 2020	Residential isolation rooms housing individuals testing positive for SARS-CoV-2, USA	Personal items, remote controls, toilets, floor, bedside table, bedrail	Non-specific (121 surface and aerosol samples)	Yes	Negative-pressure rooms (>12 ACH); negative-pressure hallways; key-card access control; unit-specific infection prevention and control (IPC) protocols including hand hygiene and changing of gloves between rooms; and PPE for staff that included contact and aerosol protection. **Viral culture method:** Vero E6 cells. CPE observed 3–4 days
Seyedmehdi 2020	Cross-sectional study Covid-19 ICU ward, Iran April 29, 2020	Not specified	10	No	Surface disinfection was performed three times a day. Air temperature 24°C, humidity 35%, air pressure 1005 mb and air velocity of 0.09 m/s. All the staff used conventional PPE.
Shin 2020	Chungbuk National University Hospital, South Korea April 2020	Bedside table, bed rail, mobile phone, tablet, call bell attached to bed, floor, door handle, sink (bathroom), toilet bowel	12	No	Mother and daughter who were COVID- positive. The most recent cleaning had occurred 4 days prior to environmental sample collection. A cycle threshold (Ct) value <40 is reported as positive.
Suzuki 2020	Cross-sectional study Cruise ship, Japan February 2020	Light switch, toilet seat, toilet floor, chair arm, TV remote, phone, table, door knob, pillow	601	Yes	Median highest and lowest temperature 13.0°C (range 6.5-18.5) and 5.5°C (0.0-9.3); median highest and lowest humidity 73 (41–98) and 40 (17–76)%. Samples collected prior to disinfection of the vessel. Case-cabins disinfected prior to sampling. Air re-circulation turned off. Subjects confined to cabins but allowed 60 mins daily walk on the deck while wearing masks and 1m social distance. **Viral culture method:** VeroE6/ TMPRSS2. CPE observation time after 4 days.
Wang 2020	Wuhan Leishenshan Hospital in Wuhan, China March 2020	ICU, treatment room, laundry room, handwashing sink, nurses' station, dialysis machine, PPE, air outlet, door handles, bed rails, dustbin, bedstand, infusion pump	62	No	7 COVID-19 patients. Negative pressure isolation ward for patients. Surfaces of objects were cleaned and disinfected 4 times/day. Diagnostic and treatment equipment were cleaned after each use.
Wang 2020a	Isolation wards in the First Affiliated Hospital of Zhejiang University, China February 2020	Isolation ICU ward and Isolation wards, including the clean area, the semi- contaminated area, and the contaminated area; front surface of N95 masks and gloves of staffs in isolation wards	45	Yes	33 laboratory-confirmed COVID-19 patients. Surfaces of objects were disinfected every 4 h in Isolation ICU ward and every 8 h in general Isolation wards. The isolation rooms were not under negative pressure. A sample was considered positive when the qRT-PCR Ct value was ≤40. **Viral culture method:** Vero-E6 cells. CPE was observed after 96 h.
Wee 2020	Dedicated isolation wards at tertiary hospital, Singapore February-May 2020	High-touch areas in the patient's immediate vicinity, toilet facilities	445	No	28 patients. Sterile premoistened swab sticks used to swab high-touch areas for 2–3 minutes over a large surface. Environmental sampling was done in the rooms to test for SARS-CoV-2 prior to terminal cleaning
Wei 2020	Non-ICU rooms in a designated isolation ward in Chengdu, China April 2020	Bedrails, room and toilet door handles, light switches, foot flush buttons, sink rims, sink and toilet bowls and drains, bedside tables, bedsheets, pillows, equipment belts on walls, floors, and air exhaust outlets	112	No	10 COVID-positive patients. Negative air pressure rooms. Rooms and toilets were cleaned and disinfected twice daily. Samples collected 4 to 7 h after the first daily cleaning.
Wei 2020a	Non-ICU isolation ward China March 2020	High-touch areas and floors in patient rooms and toilets, HCWs PPE	93	No	Surfaces cleaned/disinfected twice daily. Samples collected before the first daily cleaning. Patients had prolonged (> 30 day) SARS-CoV-2 PCR positive status for clinical samples
Wong 2020	Non-healthcare settings in Singapore February-March 2020	Accommodation rooms, toilets and elevators that have been used by COVID-19 cases	428	No	All samples were taken after the infected persons vacated the sites and have been isolated in healthcare facilities. Half of surface swabs were taken before the cleaning and disinfection and the other half was taken after the disinfection procedure. Mechanical ventilation, ambient temperature and fan-coil unit.
Wu 2020	Wuhan Hospital, China January 2020	Beeper, keyboard, computer mouse, telephone, door handle, desktop, medical equipment, bedrail, bedside table, oxygen cylinder valve, elevator button, and others such as refrigerator, IV port, and sample transfer box.	200	No	All samples were collected around 8:00 AM before routine cleaning and disinfection. HCWs used PPE. A sample was considered positive when the Ct value was ≤43.
Ye 2020	Zhongnan Medical Center in Wuhan, China February 2020	Major hospital function zones, hospital equipment/objects and medical supplies, PPE, administrative areas, and the parking lot.	626	No	Three sets of surface samples were collected using dacron swabs across major hospital function zones, hospital equipment/objects and medical supplies, and HCW's used PPE.
Yuan 2020	Hospital in Wuhan, China March 11 to March 19, 2020	High-frequency contacted surfaces in the contaminated area and the surfaces of medical staff's PPE	38	No	Samples collected 4 hours after morning disinfection of the disease area. High- flow exhaust fans on their windows and at the end of the corridor of the contaminated area to discharge the air out to the open outdoor area; natural new air inlet, to ensure that the indoor air ventilation 18 to 20 times per hour. Use of PPE by HCWs
Yung 2020	Hospital in Singapore	Bedding; the cot rail; a table situated 1 meter away from the infant's bed; and the HCW's face shield, N95 mask, and waterproof gown	6	No	Infant with COVID-19. 1 HCW. Ct values <36 were considered positive.
Zhang 2020	Hospital outdoor environment China February-March 2020	Entrance, outdoor toilet, background, in- and out-patient department	13	No	
Zhou 2020	Hospital in London, UK April 2 to 20, 2020	Bedrails, BP monitors, ward telephones, computer keyboards, clinical equipment (syringe pumps, urinary catheters), hand- cleaning facilities (hand washing basins, alcohol gel dispensers, nonpatient care areas (i.e. nursing stations and staff rooms)	218	Yes	Sampling was conducted during three tracheostomy procedures. High touch surfaces disinfected twice daily, other surfaces once daily. **Viral culture method:** Vero E6. CPE observed at 5–7 days.
Zhou 2020a	Hospital in Wuhan, China	Nosocomial surfaces, medical touching surfaces, delivery window, shoe cabinet, patient touching surfaces, clean area surfaces, hospital floor	318	No	
Zuckerman 2020	Virology Laboratory, Israel March 15th 2020	Door knobs, the outer surface of all equipment in the room, etc., with special attention to “high-touched areas	6	No	

**Table 2.** Systematic review characteristics.

Study ID	Objective	Databases searched	Search dates	Assessment of reporting quality	No. of included studies	Main results	Key conclusions
Bedrosian 2020	To assess the effectiveness of hygiene interventions against SARS- CoV-2	1. NIH COVID-19 Portfolio; 2. CDC COVID- 19 Research Articles Downloadable Database	22/01/2020 to 10/06/2020; 10/06/2020 to 10/07/2020	Not reported	35	No study assessed viral infectivity or viability, but all tested the presence or absence of SARS-CoV-2 RNA. Healthcare settings were most frequently tested (25/35, 71.4%), with households being the least tested (2/35, 5.7%). Laboratories reported the highest frequency of contaminated surfaces (20.5%, 17/ 83), while households of COVID-19 patients had the lowest frequency (2.5%, 4/161).	There is an inability to align SARS-CoV-2 contaminated surfaces with survivability data. There is a knowledge gap on fomite contribution to SARS-COV-2 transmission and a need for testing method standardization to ensure data comparability. There is a need for testing method standardization to ensure data comparability.

### Quality of included studies

**Figure 2.**
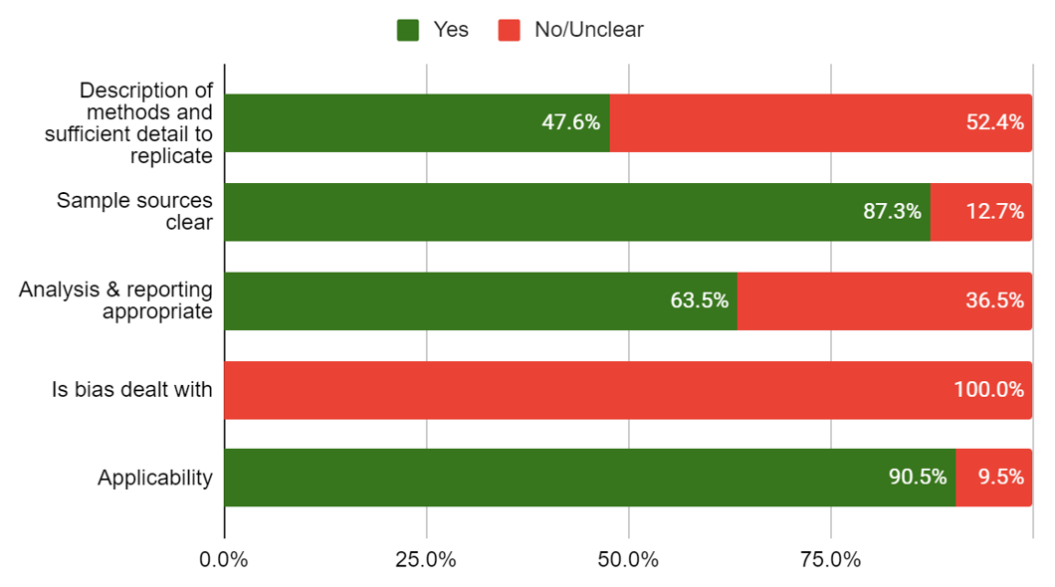
Risk of bias (n=63 primary studies).

None of the included studies were linked to or mentioned a published protocol. The risk of bias of the included studies is shown in Table 4. Less than half of the studies (47.6%) adequately reported the methods used, and none used methods to minimise bias. The overall quality of the studies was rated low to moderate (see Figure 2). 

### Reviews

We found one “systematic review” investigating the role of fomites [Bedrosian 2020] ( Table 2). The authors searched two electronic sources - articles were last downloaded on July 10, 2020. There was no published protocol, and the authors did not assess the quality of included studies. A total of 35 relevant studies were included. Over half of the studies (25/35, 75%) were conducted in healthcare settings, and four compared environmental contamination before and after standard disinfection procedures. No study assessed viral infectivity or viability, but all tested the presence or absence of SARS-CoV-2 RNA.

### Primary studies

We found 63 primary studies ( Table 1). In general, the studies did not report any hypothesis but investigated epidemiological or mechanistic evidence for fomite transmission. Forty-one studies (65.1%) were conducted in Asia, 15 (23.8%) in Europe, five (7.9%) in North America, and one each in Africa and South America (1.6% each). A total of 44 studies were conducted exclusively in hospital settings, two in hospital and quarantine facilities, three in the laboratory, and the remaining in other non-healthcare settings (public places, community, banknotes, workplace, cruise ship, quarantine rooms and hospital outdoors). Four studies were conducted exclusively in ICU and another three in ICU plus hospital wards. Five studies used before and after study design.

In 59 studies (96.7%), fomite transmission was examined in high-frequency touch surfaces ( Table 1); the remaining four studies examined circulating banknotes (1), disposable chopsticks (1) hospital staff PPE (1), and unspecified (1). The timing and frequency of sample collection and disinfection procedures were heterogeneous across studies (see Table 3). Fourteen studies (23%) performed sample collection before disinfection procedures, five studies collected samples before and after disinfection procedures, while 11 studies collected samples after disinfection. In 33 studies, the timing of sampling in relation to disinfection was not specified. In one study [Ryu 2020], disinfection procedures were not performed as required because of a lack of PPE and staff being afraid of contracting SARS-CoV-2. The number of samples per study ranged from five [Jin 2020] to 5400 [Marshall 2020].

**Table 3.** Studies sample collection characteristics.

Study ID	Frequency of sample collection	Timing of sample collection
Abrahão 2020	Not specified	Not specified
Akter 2020	NA	N/A
Amoah 2020	Twice	Unspecified
Ben-Shmuel 2020	Not specified	Not specified
Bloise 2020	Not specified	Not specified
Cheng 2020	Not specified	Not specified
Cheng 2020a	Once	Before daily disinfection
Chia 2020	Not specified	Not specified
Colaneri 2020	Not specified	Not specified
Colaneri 2020a	Once	After disinfection
D'Accolti 2020	Not specified	After disinfection
Declementi 2020	Twice	After disinfection
Ding 2020	Not specified	Before disinfection
Döhla 2020	Not specified	Not specified
Escudero 2020	Not specified	Not specified
Feng 2020	Not specified	Not specified
Fernández-de-Mera 2020	Not specified	Not specified
Ge 2020	1 to 3 times	Not specified
Guo 2020	Not specified	Not specified
Harvey 2020	Twice: Pilot phase and full-scale phase	N/A
Hu 2020	Not specified	Not specified
Hu 2020a	3 times	4 h after case confirmation
Jerry 2020	Not specified	4 h after disinfection
Jiang 2020	Not specified	Not specified
Jiang 2020a	Not specified	Before disinfection
Jin 2020	Not specified	2 h after disinfection
Kim 2020	Not specified	Not specified
Lee 2020	Not specified	After disinfection (hospital) Before disinfection (mass facilities)
Lei 2020	Twice	Before disinfection
Lui 2020	N/A	N/A
Lv 2020	Not specified	Not specified
Ma 2020	Not specified	Not specified
Maestre 2020	Not specified	Not specified
Marshall 2020	End of work shift	Before disinfection
Moore 2020	Not specified	Not specified
Nakamura 2020	Not specified	After disinfection
Nelson 2020	Not specified	Not specified
Ong 2020	5 separate time points	Before disinfection
Ong 2020a	5 days over a 2-week period	Before and after (33.3%:66.7%)
Ong 2020b	Not specified	Not specified
Pasquarella 2020	Once	After disinfection
Peyrony 2020	Not specified	Not specified
Piana 2020	Not specified	Before disinfection
Razzini 2020	Not specified	Before disinfection
Ryu 2020	Not specified	Not specified
Santarpia 2020	Not specified	Not specified
Seyedmehdi 2020	Not specified	Not specified
Shin 2020	Twice daily	After disinfection (4 days)
Suzuki 2020	Not specified	Before disinfection
Wang 2020	Not specified	Not specified
Wang 2020a	Not specified	Not specified
Wee 2020	Not specified	Before disinfection
Wei 2020	Not specified	After disinfection
Wei 2020a	Not specified	Before disinfection
Wong 2020	Not specified	Before and after (50%:50%)
Wu 2020	Not specified	Before disinfection
Ye 2020	Three sets over a 20-day period	Not specified
Yuan 2020	Not specified	After disinfection
Yung 2020	Not specified	Not specified
Zhang 2020	Not specified	Not specified
Zhou 2020	Not specified	Not specified
Zhou 2020a	Not specified	Not specified
Zuckerman 2020	Not specified	Before disinfection

**Table 4.** Quality of included studies.

Study	Description of methods and sufficient detail to replicate	Sample sources clear	Analysis & reporting appropriate	Is bias dealt with	Applicability
Abrahão 2020	Unclear	Yes	Yes	Unclear	Yes
Akter 2020	Yes	Yes	Yes	Unclear	Yes
Amoah 2020	Unclear	Yes	Yes	No	Yes
Bloise 2020	Unclear	Yes	Unclear	No	Yes
Ben-Shmuel 2020	Yes	Yes	Yes	Unclear	Yes
Cheng 2020	Unclear	Yes	Yes	No	Yes
Cheng 2020a	Unclear	Yes	Yes	Unclear	Yes
Chia 2020	No	Yes	Yes	Unclear	Yes
Colaneri 2020	Unclear	Unclear	Unclear	Unclear	Unclear
Colaneri 2020a	Yes	Yes	Unclear	Unclear	Yes
D'Accolti 2020	Yes	Yes	No	No	Yes
Declementi 2020	Unclear	Yes	Yes	Unclear	Yes
Ding 2020	Yes	Yes	Yes	Unclear	Yes
Döhla 2020	Unclear	Unclear	Yes	No	Yes
Escudero 2020	Yes	Yes	Yes	Unclear	Yes
Feng 2020	Unclear	Yes	Unclear	Unclear	Yes
Fernández-de-Mera 2020	Unclear	Yes	Unclear	No	Yes
Ge 2020	Unclear	Yes	Unclear	Unclear	Yes
Guo 2020	Unclear	Yes	Unclear	No	Unclear
Harvey 2020	Yes	Yes	Yes	Unclear	Yes
Hu 2020	No	Unclear	No	No	Unclear
Hu 2020a	Unclear	Yes	Yes	Unclear	Yes
Jerry 2020	Yes	Yes	Yes	Unclear	Yes
Jiang 2020	Yes	Yes	Unclear	Unclear	Yes
Jiang 2020a	Unclear	Yes	Yes	No	Yes
Jin 2020	Yes	Yes	Unclear	Unclear	Yes
Kim 2020	Yes	Yes	Unclear	Unclear	Yes
Lee 2020	Unclear	Yes	Yes	Unclear	Yes
Lei 2020	Unclear	Yes	Yes	No	Yes
Lui 2020	Unclear	Unclear	Unclear	Unclear	Unclear
Lv 2020	Yes	Yes	Unclear	No	Yes
Ma 2020	No	Unclear	Yes	No	Yes
Maestre 2020	Yes	Yes	Yes	Unclear	Yes
Marshall 2020	Unclear	Yes	Yes	Unclear	Yes
Moore 2020	Yes	Yes	Yes	Unclear	Yes
Nakamura 2020	Yes	Yes	Yes	Unclear	Yes
Nelson 2020	Unclear	Yes	Unclear	Unclear	Yes
Ong 2020	Yes	Yes	Yes	No	Yes
Ong 2020a	Unclear	Yes	Yes	Unclear	Yes
Ong 2020b	Unclear	Yes	Unclear	Unclear	Yes
Pasquarella 2020	Unclear	Yes	Unclear	Unclear	Yes
Peyrony 2020	Yes	Yes	Yes	Unclear	Yes
Piana 2020	Yes	Yes	Yes	Unclear	Yes
Razzini 2020	Yes	Yes	Yes	Unclear	Yes
Ryu 2020	Yes	Yes	Yes	Unclear	Yes
Santarpia 2020	Yes	Yes	Unclear	Unclear	Yes
Seyedmehdi 2020	No	Unclear	Unclear	No	Unclear
Shin 2020	Unclear	Unclear	Yes	Unclear	Yes
Suzuki 2020	Yes	Yes	Unclear	Unclear	Yes
Wee 2020	Yes	Yes	Yes	Unclear	Yes
Wei 2020	Yes	Yes	Unclear	Unclear	Yes
Wei 2020a	Unclear	Yes	Yes	Unclear	Yes
Wang 2020	Yes	Yes	Yes	Unclear	Yes
Wang 2020a	Unclear	Yes	Yes	Unclear	Yes
Wong 2020	Unclear	Yes	Yes	Unclear	Yes
Wu 2020	Unclear	Yes	Unclear	Unclear	Yes
Ye 2020	Unclear	Yes	Yes	Unclear	Yes
Yuan 2020	Yes	Yes	Yes	Unclear	Yes
Yung 2020	Unclear	Yes	Yes	No	Yes
Zhang 2020	Unclear	Unclear	Unclear	Unclear	Unclear
Zhou 2020	Yes	Yes	Yes	Unclear	Yes
Zhou 2020a	Unclear	Yes	Yes	Unclear	Yes
Zuckerman 2020	Yes	Yes	Yes	Unclear	Yes
	30	55	40	0	57
	63	63	63	63	63
	Yes	No/ Unclear			
**Description of methods and** **sufficient detail to replicate**	47.6%	52.4%			
**Sample sources clear**	87.3%	12.7%			
**Analysis & reporting** **appropriate**	63.5%	36.5%			
**Is bias dealt with**	0.0%	100.0%			
**Applicability**	90.5%	9.5%			

Eleven studies (17.5%) set out to perform viral cultures; nine of these utilised the Vero E6 cell lines method while two did not specify the method used (see Table 1). Thirteen studies (20.6%) reported cycle thresholds (Ct) for test positivity: ≤40 (8 studies); ≤43 (1 study); <35 (1 study); <36 (1 study); <37 (1 study) and <38 (1 study).

### Frequency of SARS-CoV-2 positive test

**Figure 3.**
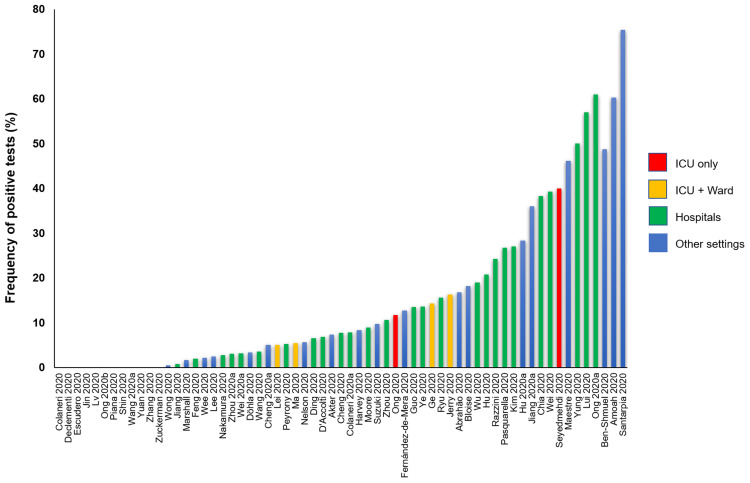
Rates of positive SARS-Cov-2 tests in studies assessing fomite transmission.

All studies reported data on the frequency of positive tests ( Table 5). ( Figure 3 shows the graphical representation of these frequencies.) The frequency of positive SARS-CoV-2 tests across 51 studies (via RT-PCR) ranged from 0.5% to 75%; 12 studies (19%) reported no positive tests. The highest frequency of positive tests was found in residential isolation rooms. Of the three studies conducted in ICU [Escudero 2000, Jin 2000, Ong 2000 and Seyedmehdi 2000], two reported positive test results (11.7% and 40%). All the four studies [Lei 2000, Ma 2000, Ge 2020, Jerry 2000] conducted in both ICU and general wards reported positive tests: 5%, 5.4%, 14.3% and 16.3%, respectively. One of the three laboratory studies [Bloise 2000] reported frequency of 18.2%; a second study [Lv 2020] reported no positive test with the conventional RT-PCR tests but reported 21.3% positivity with droplet digital PCR (ddPCR) tests; the third study [Zuckerman 2020] reported no positive tests. In a cross-sectional study of SARS-CoV-2 positive subjects confined to their cabins in a cruise ship [Suzuki 2020], the frequency of positive test was 9.7% (58/601); no positive test was detected in the non-case cabins. In one study of home quarantined subjects [Maestre 2020], 46.2% (12/26) of samples were positive for SARS-CoV-2 at two months (one month after the resolution of symptoms). In another study of two hospital patients who were SARS-CoV-2 positive [Shin 2020], no positive samples were detected after 41 days following weekly disinfection. One study conducted in a high-prevalence community setting [Döhla 2020] reported no significant association in the frequencies of positive tests between human and environmental samples (p=0.76). In all four before and after studies, there was a substantial reduction in the frequency of positive tests after surface disinfection.

**Table 5.** Findings of included studies.

Study ID	Frequency of COVID-19 positive tests	Concentration of samples	Cycle Threshold	Viral culture	Notes
Abrahão 2020	17/101 (16.8%)	70-2990 genomic units/m ^2^	Not reported	Not performed	Viral load was highest in the hospital front door ground
Akter 2020	31/425 (7.3%)	Not reported	CT values increased significantly with time on banknotes spiked with nasopharyngeal samples (p<0.05)	Not performed	Banknotes sampled from the ticket vendors and collectors at inter-city transport (bus) tested negative.
Amoah 2020	Tap handle 68.8% Toilet floor 60% Toilet seat 60% Cistern handle 60% Internal latch 53.3%	25.9 to 132.69 gc/cm ^2^	Not reported	Not performed	Viral load was consistently lower with RNA extraction versus direct detection across all sites, except with floor swab samples. No significant difference in the prevalence across sites (p ≥ 0.05). Significant differences in the concentration between the different contact surfaces (p ≤ 0.05) Use of the toilet facilities 2 to 3 times daily was observed to increase the risks of infection.
Bloise 2020	4/22 (18.2%)	Not reported	33.75 to 38.80	Not performed	qRT-PCR is unable to differentiate between infectious and non-infectious virus present on fomites
Ben-Shmuel 2020	**Symptomatic patients:**29/55 (52.7%) **Asymptomatic patients:**16/42 (38%) **Hospital isolation units** Non-ventilated patients' rooms: 9/21 (43%) Mechanically ventilated patients' rooms: 13/18 (72%) **Quarantine hotel:**16/42 (38%)	Not reported	34 to 37.9	None of the samples was culturable. No viable virus was recovered from plastic or metal coupons after 4–14 days of incubation	On viral-contaminated plastic coupons, titres of viable virus decreased by 3.5 orders of magnitude after 24 h. On metal coupons a faster reduction of 4 orders of magnitude was observed after 6 h of incubation, and similar levels of viable virus were detected at 24 h. A further decrease in viability on metal surfaces was detected at days 2 and 3.
Cheng 2020	1/13 (7.7%)	6.5 × 10 ^2^ copies/ mL of VTM	Not reported	Not performed	
Cheng 2020a	19/377 (5%)	1.1 × 10 ^2^ to 9.4 × 10 ^4^ copies/mL	Not reported	Not performed	The contamination rate was highest on patients’ mobile phones (6/77, 7.8%), followed by bed rails (4/74, 5.4%) and toilet door handles (4/76, 5.3%)
Chia 2020	Floor: 65% Bedrail: 59% Bedside locker: 47% Cardiac table: 40% Toilet seat: 18.5% ICU rooms: 0%	Not reported	28.45–35.66	Not performed	High touch surface contamination occurred in 10/15 patients (66.7%) in the first week of illness, and 3/15 (20%) beyond the first week of illness (p = 0.01). Presence of surface contamination was higher in week 1 of illness, showed some association with the Ct (P = 0.06), but was not associated with the presence of symptoms.
Colaneri 2020	0/16 (0%)	Not reported	Not reported	Not performed	
Colaneri 2020a	2/26 (7.8%)	Not reported	Not reported	None of the inoculated samples induced a cytopathic effect on day 7 of culture.	
D'Accolti 2020	Inside patients’ rooms: 3/22 (13.6%) Outside patients’ rooms: 0%	Not reported	29.54 to >35	Not performed	All samples tested positive for IC control, confirming the appropriate efficiency of the whole analysis process.
Declementi 2020	0/24 (0%)	Not reported	Not reported	Not performed	
Ding 2020	7/107 (6.5%)	407 to 723 RNA copies	36.1 to 37.9	Not performed	Positive samples were from inside door handle of the isolation rooms and toilet seat cover
Döhla 2020	4/152 (3.4 %)	Not reported	Not reported	No infectious virus could be isolated under cell culture conditions	No correlation between PCR-positive environmental samples and PCR-positive human samples, p = 0.76
Escudero 2020	0/237 (0%)	Not reported	Not reported	Not performed	
Feng 2020	Private surfaces: 4/132 (3%) Public surfaces: 0/70 (0%)	Not reported	Not reported	Could not perform viral culture due to the low virus quantity in the positive samples.	
Fernández-de- Mera 2020	7/55 (12.7%)	Not reported	36.05 to 41.06	Not performed	
Ge 2020	16/112 (14.3%)	Not reported	Not reported	Not performed	15/16 of positive samples were from ICU.
Guo 2020	***Intensive Care Unit:*** Contaminated area: 27/70 (43.5%) Semi-contaminated area: 3/33 (8.3%) Clean area: 0/12 (0%) ***General Ward:*** Contaminated area: 9/105 (8.6%) Semi-contaminated area: 0/24 (0%) Clean area: 0/46 (0%)	ICU Contaminated area: 1.5 × 10 ^5^ to 7.1 × 10 ^3^ copies/ sample NA, not applicable; ND, not determined for other sites	Not reported	Not performed	The rate of positivity was higher for surfaces frequently touched by medical staff or patients. The highest rates were for computer mice (ICU 6/8, 75%; GW 1/5, 20%), followed by trash cans (ICU 3/5, 60%; GW 0/8), sickbed handrails (ICU 6/14, 42.9%; GW 0/12), and doorknobs (GW 1/12, 8.3%).
Harvey 2020	29/348 (8.3%)	Majority of our positive samples not quantifiable. 2.54 to 102.53 gc/cm ^2^	Not reported	Not performed	The estimated risk of infection from touching a contaminated surface was low (less than 5 in 10,000). The percent of positive samples per week was inversely associated with daily maximum temperature (p=0.03) and absolute humidity (p=0.02). Temperature was inversely correlated with COVID-19 case numbers (p=0.01).
Hu 2020	5/24 (20.8%)	Viral RNA ranged from 1.52 × 10 ^3^ to 4.49 × 10 ^3^ copies/ swab	Not reported	Not performed	
Hu 2020a	1st batch: 11/23 (47.8%) 2nd batch: 2/23 (8.7%)	Not reported	26 to 39	Not performed	70% of samples taken from the bedroom tested positive for SARS-CoV-2, followed by 50% of samples taken from the bathroom and that of 33% from the corridor. The inner walls of toilet bowl and sewer inlet were the most contaminated sites with the highest viral loads.
Jerry 2020	COVID-19 patient room: 11/26 (42.3%) Post-disinfection: 1/25 (4%) Nurses station: 1/29 (3.4%)	Not reported	Not reported	Not performed	
Jiang 2020	1/130 (0.8%)	Not reported	Not reported	Not performed	
Jiang 2020a	8/22 (36%)	Not reported	28.75 to 37.59	Not performed	All control swab samples were negative for SARS-CoV-2 RNA.
Jin 2020	0/5 (0%)	Not reported	Not reported	Not performed	
Kim 2020	All surfaces: 89/320 (27%) Rooms without routine disinfection: 52/108 (48%) Rooms with routine disinfection: 0%	Not reported	Ct values varied across rooms: ≤ 35; > 35 and ≤ 40	Not performed	
Lee 2020	Hospitals: 0/68 (0%) Mass facilities: 2/12 (16.7%)	Not reported	27.4 to 34.8	Not performed	Note: Hospitals were disinfected.
Lei 2020	9/182 (5%)	Not reported	Patient's facemask (Ct = 38.6) Floor of a patient's room (Ct = 42.4 and 41.2) Patient's mobile phones (Ct = 44.1 and 41.0)	Not performed	
Lui 2020	8/14 (57%)	3.4 × 10 ^3^ copies/ mL	Not reported	Not performed	The concentration of SARS-CoV-2 RNA detected from chopsticks was significantly lower than those of nasopharyngeal swabs and sputum samples, p<0.001
Lv 2020	qRT-PCR: 0%; ddPCR: 13/61 (21.3%)	From 0.84 copies/cm ^2^ to 37.4 copies/cm ^2^	Not reported	Not performed	
Ma 2020	All surfaces: 13/242 (5.4%) Object handles: 0/26 (0%)	Not reported	36.38 ± 1.92	Not performed	
	13/242 (5.4%)		33.5 to 39.54	Not performed	
Maestre 2020	12/26 (46.2%)	20 copies/cm ^2^ in master bedroom used by both occupants	Not reported	Not performed	The highest SARS-CoV-2 RNA signal was observed on the top of the TV surface. The surfaces in the bathroom did not yield any SARS-CoV-2 signal, except for the toilet handle.
Marshall 2020	Locations with positive employees: 1.7% Locations without positive employees: 0.13%	Not reported	35 to 38	Not performed	Locations with positive environmental surfaces had 10 times greater odds (P≤0.05) of having positive employees compared to locations with no positive surfaces.
Moore 2020	30/336 (8.9%)	2·2 × 10 ^5^ to 59 genomic copies/ swab	28·8 to 39·1	No CPE or a decrease in Ct values across the course of three serial passages were observed suggesting the samples did not contain infectious virus	
Nakamura 2020	4/141 (2.8%)	2.96 × 10 ^3^ copies/ swab to 4.78 × 10 ^3^ copies/swab	Not reported	Not performed	
Nelson 2020	All surfaces: 5/89 (5.6%) BP cuffs: 5/9 (44%)	Not reported	37.38 to 39.18	Not performed	
Ong 2020	ICU ward common areas: 6/60 (10%) Staff pantry: 2/15 (13.3%)	Not reported	Not reported	All samples in common areas and staff pantry were negative on viral cell culture.	Viral cell culture was not attempted on patient room samples due to resource limitations.
Ong 2020a	Environmental sites: 17/28 (61%) PPE: 1/10 (10%) Post-disinfection: 0%	Not reported	30.64 to 38.96	Not performed	
Ong 2020b	0/90 (0%)	Not reported	20.8 to 32.23	Not performed	
Pasquarella 2020	4/15 (26.7%)	Not reported	31 to 35	Not performed	
Peyrony 2020	10/192 (5.2%)	Not reported	35.71 to 39.69	Because of weak amounts of viral RNA in positive samples, there was no attempt to isolate viruses in cell culture	
Piana 2020	0/96 (0%)	Not reported	Not reported	Not performed	
Razzini 2020	9/34 (24.3%)	Not reported	21.5 to 24	Not performed	
Ryu 2020	Hospital A: 10/57 (17.5%) Hospital B: 3/22 (13.6%)	Not reported	Not reported	Not performed	Hospital A (more severe patients in well- equipped isolation rooms) Hospital B (less severe patients in common hospital rooms)
Santarpia 2020	All personal items: 70.6% Toilets: 81.0% Room surfaces: 75.0% Cellular phones: 77.8% Bedside rails and tables: 75% Window ledges: 72.7%	Mean concentration ranged from 0.17 to 0.82 copies/µL across surfaces tested	Not reported	Due to the low concentrations recovered in these samples cultivation of virus was not confirmed	
Seyedmehdi 2020	4/10 (40%)	3227 ± 3674 copies/mL	Not reported	Not performed	
Shin 2020	0/12 (0%)	Not reported	27.97 to 39.78	Not performed	
Suzuki 2020	58/601 (9.7%)	Not reported	26.21-38.99	No virus was cultured	SARS-CoV-2 RNA was detected from about two-thirds of all case-cabins swabbed, while it was not detected from any non-case cabins.
Wang 2020	ICU: 2/28 (7.1%) General ward: 0/34 (0%)	Not reported	37.56 and 39.00	Not performed	
Wang 2020a	0/45 (0%)	No positive samples	No positive samples	No positive samples	
Wee 2020	10/445 (2.2%)	Not reported	32.69	Not performed	Of the 4 index cases who required supplemental oxygen in the general ward, 75.0% (3/4) had positive environmental surveillance samples for SARS-CoV-2, compared with 8.2% (2/24) among those not on supplemental oxygen (P = 0.01)
Wei 2020	44/112 (39.3%)	Not reported	Not reported	Not performed	
Wei 2020a	3/93 (3.2%)	Not reported	17.5 to 32.9	Not performed	
Wong 2020	Before disinfection: 2/428 (0.5%) Post-disinfection: 0%	Not reported	Not reported	Not performed	
Wu 2020	38/200 (19%)	Not reported	Not reported	Not performed	
Ye 2020	85/626 (13.6%)	Not reported	Not reported	Not performed	The most contaminated objects were self- service printers (20%), desktops/keyboards (16.8%), and doorknobs (16%). Hand sanitizer dispensers (20.3%) and gloves (15.4%) were the most frequently contaminated PPE.
Yuan 2020	0/38 (0%)	Not reported	Not reported	Not performed	
Yung 2020	Environmental sites: 3/3 (100%) PPE: 0/3 (0%)	Not reported	28.7, 33.3, and 29.7	Not performed	
Zhang 2020	0/13 (0%)	Not reported	Not reported	Not performed	
Zhou 2020	23/218 (10.6%)	10 ^1^ to 10 ^4^ genome copies per swab	>30.	No virus was cultured	Viral RNA was detected on 114/218 (52.3%) of all surfaces and 91/218 (41.7%) of "suspected" surfaces
Zhou 2020a	10/318 (3.1%)	3–8 viruses/cm ^2^	Not reported	Not performed	
Zuckerman 2020	0/6 (0%)	Not reported	Not reported	Not performed	

### Viral load and concentration

A total of 17 studies reported data on viral concentration ( Table 5); the units of measure used to report this data varied across the studies and included genomic copies/swab (4 studies), genomic copies/cm ^2^ (4 studies), genomic copies/mL (4 studies), and 1 study each for mean concentration, viruses/cm ^2^, genomic units/m ^2^, genomic copies/sample and RNA copies. We found it impossible to make any comparisons across the studies because of the heterogeneity in units of measurement.

### Cycle thresholds

A total of 28 studies (44.4%) reported data in Ct with values ranging from 20.4 to 44.1 ( Table 5). One study of ICU patients [Razzini 2020] reporting positive rates of 24.3% (9/34) had the lowest range of Ct (21.5-24), while another study of ICU and isolation ward patients [Lei 2020] reporting positive rates of 5% (9/182) had the highest range of Ct (38.6-44); in both studies, the Ct for positivity was ≤40.

### Viral culture

Of the 11 studies that planned to perform viral culture, only two (18.2%) reported Ct values that could act as prompts to undertake viral isolation ( Table 6). Only two studies provided information on the timing of sample collection for viral culture but were missing key details with respect to collection related to the timing of the onset of symptoms of the patients with respect to the collection and timing. One study of subjects in a cruise ship [Suzuki 2020] reported collecting samples for viral culture from 1–17 days after the cabin was vacated on a cruise ship and at least 17 days after the quarantining to cabins was ordered and 8 days after the first cabin cleaning, while another study of patients in residential isolation [Santarpia 2020] reported collecting the samples on “days 5–9” or “day 10” of occupancy at a medical centre or quarantine unit, all of whom were evacuated from the same cruise ship reported previously and would have been at least 2 weeks from the last day of quarantine [Suzuki 2020]. The incubation period ranged from 4–7 days and there were subtle differences in the culture media used across the studies ( Table 6). None of the studies reported success with viral culture despite positive RT-PCR detection tests. There were methodological issues with the techniques employed for viral culture across the studies (see Table 6).

**Table 6.** Findings of included studies: viral culture.

Study ID	Threshold for viral culture	Timing of viral culture	Method used for viral culture	Cycle Threshold	Results of viral culture
Ben- Shmuel 2020	Not specified	Not specified	Applied 200 μL from 10-fold serial sample dilutions upon VERO E6 cell cultures in 24-well plates. After 1 h, wells were overlaid with 1 mL of MEM medium supplemented with 2% foetal calf serum (FCS), MEM non-essential amino acids, 2 mM L-glutamine, 100 units/mL penicillin, 0.1% streptomycin, 12.5 units/mL nystatin and 0.15% sodium bicarbonate. Cells were incubated for 5 days (37°C, 5% CO2), and CPEs were observed after fixation with crystal violet solution.	34 to 37.9	None of the samples was culturable. No viable virus was recovered from plastic or metal coupons after 4–14 days of incubation
Colaneri 2020a	All 26 samples were inoculated onto susceptible Vero E6 cells	Not specified	A 200-μL sample was inoculated onto a Vero E6 confluent 24-well microplate for virus isolation. After 1 hour of incubation at 33°C in 5% CO2 in air, the inoculum was discarded and 1 mL of medium for respiratory viruses was added (Eagle's modified minimum essential medium supplemented with 1% penicillin, streptomycin and glutamine, and 5 mg/mL trypsin) to each well. Cells were incubated at 33°C in 5% CO2 in air and observed by light microscopy every day for cytopathic effect. After a 7-day incubation, 200 μL of supernatant was used for molecular assays.	Not reported	None of the inoculated samples induced a cytopathic effect on day 7 of culture.
Döhla 2020	Not specified	Not specified	Seeded Vero E6 cells in 24 well plates or T25 flasks at a density of 70–80 %. Cells were incubated with 200µl (24 well) – 1000 µl (T25 flask) of the sample material supplemented with 1x penicillin/streptomycin/amphotericin B and incubated for 1 h at 37°C in 5 % CO2. For water samples, 10% (v/v) of inoculation volume was replaced by 10xPBS to obtain a final concentration of 1xPBS. After 1 h of incubation, the inoculum was removed, Dulbecco’s Modified Eagle’s medium (Gibco) with 3 % foetal bovine serum (Gibco) and 1x penicillin/streptomycin/ amphotericin B was added. Cells were incubated over several days at 37°C, 5% CO2 and observed for development of a cytopathic effect that typically occurs for growth of SARS-CoV-2 on Vero E6 cells.	Not reported	No infectious virus could be isolated under cell culture conditions from any sample
Feng 2020	Not specified	Not specified	Not reported	Not reported	Could not perform viral culture due to the low virus quantity in the positive samples.
Moore 2020	<34	Not specified	Vero E6 cells (Vero C1008; ATCC CRL-1586) in culture medium [MEM supplemented with GlutaMAX-I, 10% (v/v) fetal bovine serum (FBS), 1X (v/v) non-essential amino acids and 25 mM HEPES] were incubated at 37oC. Cells (1 x 106 cells/25 cm2 flask) were washed with 1X PBS and inoculated with ≤1 mL environmental sample and incubated at 37°C for 1 h. Cells were washed with 1X PBS and maintained in 5 mL culture medium (4% FBS) with added antibiotic– antimycotic (4X), incubated at 37°C for 7 days and monitored for cytopathic effects (CPE). Cell monolayers that did not display CPE were subcultured up to three times, providing continuous cultures of ~30 days.	28·8 to 39·1	No CPE or a decrease in Ct values across the course of three serial passages were observed suggesting the samples did not contain infectious virus
Ong 2020	Positive swabs from PCR	Not specified	Monolayers of Vero C1008 cells (ATCC-1586) in T25 flasks were inoculated with 1 mL inoculum (500 µL of the swab sample and 500 µL of Eagle’s MEM) and cultured at 37°C, 5% CO2 with blind passage every 7 days. Also, 140 µL cell culture was used for RNA extraction and real-time PCR twice per week to monitor changes in target SARS-CoV-2 genes as an indication of successful viral replication. In the absence of CPEs and real-time PCR indication of viral replication, blind passages continued for a total of 4 passages before any sample was determined to be negative of viable SARS-CoV-2 virus particles.	Not reported	All samples in common areas and staff pantry were negative on viral cell culture.
Peyrony 2020	Not specified	Not specified	Not specified	35.71 to 39.69	Because of weak amounts of viral RNA in positive samples, there was no attempt to isolate viruses in cell culture
Santarpia 2020	Subset of samples that were positive for viral RNA by RT-PCR	Days 5–9 of patient occupancy for one site and day 10 occupancy for the second site. No information is provided on the date of onset of patient symptoms	Vero E6 cells. Several indicators were utilized to determine viral replication including cytopathic effect (CPE), immunofluorescent staining, time course PCR of cell culture supernatant, and electron microscopy.	Not reported	Cultivation of virus on cell culture was not confirmed including the air sample.
Suzuki 2020	Some samples from which viral RNA was present	No details provided from time of symptom onset but ranged from 1–17 days after the cabin was vacated and at least 17 days after the quarantining to cabins was ordered and 8 days after the first cabin cleaning	Samples were mixed with Dulbecco’s modified Eagle medium supplemented with typical concentrations of penicillin G, streptomycin, gentamicin, amphotericin B and 5% fetal bovine serum. They were inoculated on confluent VeroE6/TMPRSS2 cells. Culture medium at 0- or 48-hours post-infection (hpi) were collected and diluted10-fold in water, then boiled for 5 minutes. CPE observation after 4 days.	26.21-38.99	No virus was cultured
Wang 2020a	Not specified	Not specified	Samples were obtained and inoculated on Vero-E6 cells for virus culture. The cytopathic effect (CPE) was observed after 96 h.	No positive samples	No positive samples
Zhou 2020	Ct value <30	Not specified	Vero E6 and Caco2 cells were used to culture virus. The cells were cultured in DMEM supplemented with heat inactivated fetal bovine serum (10%) and Penicillin/Streptomycin (10, 000 IU/mL &10, 000 µg/mL). For propagation, 200 µL of samples were added to 24 well plates. After 5–7 days, cell supernatants were collected, and RT-qPCR to detect SARS-CoV-2 performed. Samples with at least one log increase in copy numbers for the E gene (reduced Ct values relative to the original samples) after propagation in cells were considered positive.	>30.	No virus was cultured

## Discussion

We found 63 primary studies investigating the role of fomites in SARS-CoV-2 transmission. The results of the majority of these studies show that SARS-CoV-2 RNA can be frequently detected on surfaces in both healthcare and non-healthcare settings. However, there were no positive culture results for studies that attempted to culture for viable virus. There is a wide variation in study setting and designs across studies, and the overall quality of published studies is low to moderate. The heterogeneity in study design and methodology makes it difficult to compare results across studies. The results of the systematic review (n=35) [Bedrosian 2020] showed that surface contamination was greatest in laboratories and least in households; however, none of the included studies addressed viral infectivity. The review authors did not assess the reporting quality of the primary studies and the search periods are now outdated.

The inability to culture the virus despite positive PCR detection tests may indicate either that surface contamination does not support viral growth and hence transmissibility or that the timing of collection was at a point of time where no viable virus would be likely to be found. The latter possibility is considered highly likely on the basis of culturing without the benefit of looking at the timing of the specimens with respect to symptom onset in the patients or evaluating Ct values to ensure they were very low to enhance the likelihood of finding viable virus or culturing attempts from samples with relatively high Ct values where it would be exceedingly difficult to culture viable virus. The substantial reduction in positive detection rates before and after studies (and in some ICU settings) suggests that good hygiene procedures can minimise the risk of surface contamination. The inconsistency in describing *a priori* Ct values across the studies, coupled with the wide range in actual Ct values, suggests that the reported positive SARS-CoV-2 RNA detection rates are markers of previous viral presence from non-viable virus.

In a systematic review assessing the role of fomites in virus transmission in the Middle East Respiratory Syndrome (MERS) ^11^, the authors reported possible evidence of fomite contamination but the evidence for fomite transmission was anecdotal. Our review findings are consistent with these observations. In an observational study of four hospitalised patients with MERS ^12^, there was positive viral culture from fomites including bed sheets, bed rails, intravenous fluid hangers, and radiograph devices. In contrast to that study, published research on SARS-CoV-2 shows no evidence of positive viral culture to date. Our review findings support several national and international guidelines recommending good hygiene practices to reduce the spread of SARS-CoV-2 ^13– 15^.

We identified one non-peer-reviewed (pre-print) systematic review that assessed fomite contamination in SARS-CoV-2 ^16^. The authors concluded that the quality of measurements was poor, and the reliability of the data is uncertain. Our findings are consistent with these. Compared to that review, we searched more databases, included more than twice the number of included studies, and accounted for the reporting quality of included studies.

Although there has been much research into fomite transmission of SARS-CoV-2, much uncertainty remains, and it is difficult to draw meaningful conclusions. Firstly, the variation in Ct across the studies suggests that there is no standardized threshold for detection of SARS-CoV-2 RNA. Some studies have shown that lower Ct correlates with higher genomic load ^17^.

The studies included in this review used Ct of <35 to <43; these threshold values indicate that some of the positive tests reported in the studies may be misleading. Future research aimed at establishing internationally accepted Ct values should be considered a priority. The discrepancies in units of measurements for viral load and/or concentration also creates confusion. Therefore, standardized checklists for reporting of studies investigating SARS-CoV-2 transmission should be developed, including mandatory publishing of protocols, including the timing of the collection of any environmental specimens with respect to patient symptom onset. Looking for viable virus long after a patient has developed a significant innate and adaptive immunologic response will consistently yield negative results.

That all 11 culture studies failed to isolate the virus with significant fundamental methodological flaws indicates that the threshold for transmissibility from contaminated surfaces is unknown and more rigorous and carefully orchestrated studies are required before any conclusions may be drawn. One factor likely relates to the timing of sample collection after the onset of infection. Two studies reported the timeframe for sample collection but without precision while nine did not report any timelines. The mean incubation period of SARS-CoV-2 is 5–6 days ^2^; therefore, sample collection within the first few days of infection onset is likely to yield greater viral RNA load and result in better infectivity and culture results. Future studies should endeavour to collect surface samples of likely contaminated surfaces and medical equipment within useful timeframes and should also report this variable with their results.

As reported in the results, findings from one study [Lv 2020] showed that detection rates were different when qRT-PCR was compared with ddPCR. Interestingly, the authors of another included study [Bloise 2020] concluded that qRT-PCR is unable to differentiate between infectious and non-infectious viruses. Therefore, the use of RT-PCR as the gold standard for detection of SARS-CoV-2 requires further research. The positive findings from the before and after studies show that good hygiene procedures should continue to be a cornerstone for the management of SARS-CoV-2 and other communicable diseases.

### Strengths and limitations

To our knowledge, this is the most comprehensive review to date that evaluates the role of fomites in SARS-CoV-2 transmission. We extensively searched the literature for published studies and included studies that are yet to undergo peer review. We also accounted for the quality of the studies and have presented summary data for some subgroups where possible. However, we recognize several limitations. We may not have identified all published studies investigating the role of fomites; indeed, several other studies may have been published after the last search date for this review. Heterogeneity due to variations in study designs and lack of uniformity in measurement metrics prevent us from statistically combining data across studies and limits the validity and applicability of the review results.

## Conclusion

The evidence from published research suggests that SARS-CoV-2 RNA can be readily detected on surfaces and fomites. There is no evidence of viral infectivity or transmissibility via fomites to date but no studies to date have been found to be methodologically robust and of high enough quality to even adequately address the question. Good hygienic practices appear to reduce the incidence of surface contamination. Published studies are heterogeneous in design, methodology and viral reporting metrics and there are flaws in the reporting quality. Standardized guidelines for the design and reporting of research on fomite transmission should now be a priority.

## Data availability

### Underlying data

All data underlying the results are available as part of the article and no additional source data are required.

### Extended data

Figshare: Extended data: SARS-CoV-2 and the Role of Fomite Transmission: A Systematic Review, https://doi.org/10.6084/m9.figshare.14247113.v1 ^9^.

This project contains the following extended data:

- Appendix 1: Protocol
- Appendix 2: Search Strategy
- Appendix 3: List of excluded studies
- Appendix 4: References to included studies

### Reporting guidelines

Figshare: PRISMA checklist for ‘SARS-CoV-2 and the Role of Fomite Transmission: A Systematic Review’, https://doi.org/10.6084/m9.figshare.14247113.v1 ^9^.

Data are available under the terms of the Creative Commons Attribution 4.0 International license (CC-BY 4.0).
