# Supplementary material for: SARS-CoV-2 and the role of fomite transmission: a systematic review
Source: F1000Res. 2021 Jun 14;10:233. Originally published 2021 Mar 24. [Version 3] doi: 10.12688/f1000research.51590.3 (PMC8176266; doi:10.12688/f1000research.51590.3)
Supplement: Peer review history

# Peer review: Reviewer response for version 2

**DOI:** 10.5256/f1000research.56906.r86160

**Published:** 2021-06-03

**Authors:** Ana Karina Pitol Garcia

**Affiliations:** 1 Department of Civil and Environmental Engineering, Imperial College London, London, UK

The authors have addressed most of the major concerns.

 I still believe that it's not clear what the authors mean by "bias". The authors used circular reasoning in their explanation; they state that they assessed "bias" by asking if the reviewed articles acknowledged "bias", but they never defined the term or mentioned the exact questions used to determine it.  

 Nevertheless, this article provides a good summary of the scientific publications that have survey SARS-CoV-2 on surfaces and is, therefore, a valuable contribution.

Are the rationale for, and objectives of, the Systematic Review clearly stated?

Yes

Is the statistical analysis and its interpretation appropriate?

Not applicable

Are sufficient details of the methods and analysis provided to allow replication by others?

Partly

Are the conclusions drawn adequately supported by the results presented in the review?

Partly

Reviewer Expertise:

Disease transmission, Quantitative Microbial Risk Assessment (QMRA), fomite-mediated transmission, virus transfer.

I confirm that I have read this submission and believe that I have an appropriate level of expertise to confirm that it is of an acceptable scientific standard.

# Peer review: Reviewer response for version 2

**DOI:** 10.5256/f1000research.56906.r86161

**Published:** 2021-06-03

**Authors:** Emanuel Goldman

**Affiliations:** 1 Department of Microbiology, Biochemistry and Molecular Genetics, New Jersey Medical School, Newark, NJ, USA

I'm still not entirely convinced that the "bias" parameter included in the analysis is useful, since there is some element of subjectivity in making that assessment. Nevertheless, the authors have given a fuller accounting of what's involved in this parameter, so at least, a reader can draw their own conclusions regarding alleged "bias".

 Overall, the authors have appropriately responded to my comments and suggestions, and I am satisfied that this manuscript is now acceptable as a peer-reviewed publication and a useful contribution to the literature.

Are the rationale for, and objectives of, the Systematic Review clearly stated?

Yes

Is the statistical analysis and its interpretation appropriate?

Yes

Are sufficient details of the methods and analysis provided to allow replication by others?

Partly

Are the conclusions drawn adequately supported by the results presented in the review?

Yes

Reviewer Expertise:

Microbiology, Virology

I confirm that I have read this submission and believe that I have an appropriate level of expertise to confirm that it is of an acceptable scientific standard.

# Peer review: Reviewer response for version 1

**DOI:** 10.5256/f1000research.54776.r83869

**Published:** 2021-05-11

**Authors:** Ana Karina Pitol Garcia

**Affiliations:** 1 Department of Civil and Environmental Engineering, Imperial College London, London, UK

The authors compiled and summarized many scientific publications that surveyed SARS-CoV-2 on surfaces. Surface contamination is an essential piece of information needed to understand the role of fomites in disease transmission. The authors collected data on the presence of RNA and viable SARS-CoV-2. Notable, out of the 11 identified papers that attempted viral culture, none fund cytopathic effects. Therefore, no viable SARS-CoV-2 has been recovered from surfaces up to date. The review is comprehensive, timely, and valuable for understanding the relative contribution of fomite-mediated transmission to the spread of SARS-CoV-2.

**Major comments:**

**Methods**

 The authors mention using an adapted version of "QUADAS-2 criteria" to assess the quality of the studies, but the exact methodology is not reported neither in the main article nor in the extended data. More transparency is needed regarding the exact questions used to determine quality and bias.

 Additionally, the authors criticize publications that do not report "patient information" such as "symptom onset in the patients". Nevertheless, this information is not relevant in settings with no COVID-19 patients (surfaces at gas stations, bus stations, hospital wards, etc.).

**Discussion**

 Regarding the following statement:

*"The inability to culture the virus despite positive PCR detection tests may indicate either that surface contamination does not support viral growth and hence transmissibility or that the timing of collection was at a point of time where no viable virus would be likely to be found."*

 The first hypothesis is not clear, *"The inability to culture the virus despite positive PCR detection tests may indicate either that surface contamination does not support viral growth and hence transmissibility" - *We know that the virus needs a host cell to replicate. The capacity of fomites to serve as a vehicle for the transmission of SARS-CoV-2 depends on factors such as the decay rate of the virus on the surface and on the hands, the virus transfer rate (surface to hand, and hand to face), the frequency of touch between the hands and face, the dose-response curve of the virus, among other parameters. Even if the virus does not "grow" on the surface, there is still potential for transmission. Therefore, this sentence needs to be removed or clarified.

 Second hypothesis: *"The inability to culture the virus despite positive PCR detection tests may indicate … that the timing of collection was at a point of time where no viable virus would be likely to be found." - *The authors suggest throughout the discussion that the 11 studies that cultured the virus showing not cytopathic effects have *"significant fundamental methodological flaws"* (which are not clearly pointed out in the manuscript), directing the reader to conclude that this is probably the reason why no attempt at culturing viruses from surfaces has been successful. It's helpful to point out that SARS-CoV-2 RNA is much more stable on fomites than infective SARS-CoV-2 virus, and it is found in much greater concentrations. For example, in an experimental study where SARS-CoV-2 was inoculated on surfaces, Paton *et al. *(2021) ^1^ found that the number of RNA recovered from the surfaces was 10 ^3^ to 10 ^8^ times higher than the number of viable virus in the samples. Given the relatively low concentration of RNA found on surfaces in the compiled articles, it is expected that the 11 studies that attempted to culture the virus could not find cytopathic effects. It is true, as the authors point out, that the transmission via fomites cannot be discarded. Nevertheless, the evidence that the authors compiled can already give an idea of how unlikely fomite-mediate transmission is in most settings.

**Minor comments:**

**Abstract**

 Please, clarify the statement *"SARS-CoV-2 has been detected in fomites..."* by replacing it with "SARS-CoV-2 RNA has been detected in fomites..."

**Introduction**

 The statement, *"it has been reported that SARS-CoV-2 can be transmitted indirectly through fomites or surfaces."* is not supported by the reference. The reference only suggests the possibility of fomite transmission based on (1) the presence of SARS-CoV-2 RNA on surfaces, and (2) experimental evidence of virus survival at specific conditions and high concentrations. Up to date, no study has "reported" fomite-mediated transmission. Replacing the word "reported" by "suggested" would be better.

**Discussion**

 In the following sentence, *"We identified one non-peer-reviewed (pre-print) systematic review that assessed fomite contamination in SARS-CoV-2."* perhaps what the authors intended to say was, "We identified one non-peer-reviewed (pre-print) systematic review that assessed SARS-CoV-2 contamination in fomites."?

**Figure 3**

 It would be more informative for the reader if the plot could distinguish between public spaces and rooms with COVID-19 patients. The authors could break the "other" category and include "isolation or quarantine room", since the most frequently contaminated spaces were quarantine rooms.

Are the rationale for, and objectives of, the Systematic Review clearly stated?

Yes

Is the statistical analysis and its interpretation appropriate?

Not applicable

Are sufficient details of the methods and analysis provided to allow replication by others?

Partly

Are the conclusions drawn adequately supported by the results presented in the review?

Partly

Reviewer Expertise:

Disease transmission, Quantitative Microbial Risk Assessment (QMRA), fomite-mediated transmission, virus transfer.

I confirm that I have read this submission and believe that I have an appropriate level of expertise to confirm that it is of an acceptable scientific standard, however I have significant reservations, as outlined above.

## References

[1] : Persistence of SARS-CoV-2 virus and viral RNA on hydrophobic and hydrophilic surfaces and investigating contamination concentration. *bioRxiv* .2021; 10.1101/2021.03.11.435056 10.1101/2021.03.11.435056

# Response

**Published:** 2021-05-14

**Authors:** IGHO ONAKPOYA

**Affiliations:** University of Oxford, UK

**Peer reviewer's comment:**

 The authors compiled and summarized many scientific publications that surveyed SARS-CoV-2 on surfaces. Surface contamination is an essential piece of information needed to understand the role of fomites in disease transmission. The authors collected data on the presence of RNA and viable SARS-CoV-2. Notable, out of the 11 identified papers that attempted viral culture, none fund cytopathic effects. Therefore, no viable SARS-CoV-2 has been recovered from surfaces up to date. The review is comprehensive, timely, and valuable for understanding the relative contribution of fomite-mediated transmission to the spread of SARS-CoV-2.

**Authors' response: **Thank you.

**Peer reviewer's comment:**

**Major comments:**

**Methods**

 The authors mention using an adapted version of "QUADAS-2 criteria" to assess the quality of the studies, but the exact methodology is not reported neither in the main article nor in the extended data. More transparency is needed regarding the exact questions used to determine quality and bias.

**Authors' response: **Now reads thus: “We assessed the risk of bias using five domains from the QUADAS-2 criteria [9]; we adapted this tool because the included studies were not designed as diagnostic accuracy studies. The domains assessed were: (i) study description - was there sufficient description of methods to enable replication of the study? (ii) sample sources – was there a clear description of sample sources? (iii) description of results - was the reporting of study results and analysis appropriate? (iv) risk of bias - did the authors acknowledge any potential biases, if yes were any attempts made to address these biases? (v) applicability – is there any concern that the interpretation of test results differs from the study question? For each bias domain, the risk was judged as “low”, “unclear” or “high”.”

**Peer reviewer's comment:**

 Additionally, the authors criticize publications that do not report "patient information" such as "symptom onset in the patients". Nevertheless, this information is not relevant in settings with no COVID-19 patients (surfaces at gas stations, bus stations, hospital wards, etc.).

**Authors' response: **Now reads thus: “The latter possibility is considered highly likely on the basis of culturing without the benefit of looking at the timing of the specimens with respect to symptom onset (in study settings that had SARS-CoV-2 positive patients) …”

**Peer reviewer's comment:**

**Discussion**

 Regarding the following statement:

*"The inability to culture the virus despite positive PCR detection tests may indicate either that surface contamination does not support viral growth and hence transmissibility or that the timing of collection was at a point of time where no viable virus would be likely to be found."*

 The first hypothesis is not clear, *"The inability to culture the virus despite positive PCR detection tests may indicate either that surface contamination does not support viral growth and hence transmissibility" - *We know that the virus needs a host cell to replicate. The capacity of fomites to serve as a vehicle for the transmission of SARS-CoV-2 depends on factors such as the decay rate of the virus on the surface and on the hands, the virus transfer rate (surface to hand, and hand to face), the frequency of touch between the hands and face, the dose-response curve of the virus, among other parameters. Even if the virus does not "grow" on the surface, there is still potential for transmission. Therefore, this sentence needs to be removed or clarified.

**Authors' response: **We have revised the statement and added a reference. Now reads thus: “The inability to culture the virus despite positive PCR detection tests suggests that SARS-CoV-2 RNA is more stable (and likely found in greater concentrations) on fomites than infective SARS-CoV-2 virus [11]”. Factors known to affect the ability of fomites to serve as transmitters of respiratory viruses include the rate of decay of the virus on the surface and on the hands, the virus transfer rate (surface to hand, and hand to face), the frequency of touch between the hands and face, the dose-response curve of the virus, temperature and humidity, amongst others [12].”

**Peer reviewer's comment:**

 Second hypothesis: *"The inability to culture the virus despite positive PCR detection tests may indicate … that the timing of collection was at a point of time where no viable virus would be likely to be found." - *The authors suggest throughout the discussion that the 11 studies that cultured the virus showing not cytopathic effects have *"significant fundamental methodological flaws"* (which are not clearly pointed out in the manuscript), directing the reader to conclude that this is probably the reason why no attempt at culturing viruses from surfaces has been successful. It's helpful to point out that SARS-CoV-2 RNA is much more stable on fomites than infective SARS-CoV-2 virus, and it is found in much greater concentrations. For example, in an experimental study where SARS-CoV-2 was inoculated on surfaces, Paton *et al. *(2021) ^1^ found that the number of RNA recovered from the surfaces was 10 ^3^ to 10 ^8^ times higher than the number of viable virus in the samples. Given the relatively low concentration of RNA found on surfaces in the compiled articles, it is expected that the 11 studies that attempted to culture the virus could not find cytopathic effects. It is true, as the authors point out, that the transmission via fomites cannot be discarded. Nevertheless, the evidence that the authors compiled can already give an idea of how unlikely fomite-mediate transmission is in most settings.

**Authors' response: **We have revised the statement.

**Peer reviewer's comment:**

**Minor comments:**

**Abstract**

 Please, clarify the statement *"SARS-CoV-2 has been detected in fomites..."* by replacing it with "SARS-CoV-2 RNA has been detected in fomites..."

**Authors' response: **Added “RNA”

**Peer reviewer's comment:**

**Introduction**

 The statement, *"it has been reported that SARS-CoV-2 can be transmitted indirectly through fomites or surfaces."* is not supported by the reference. The reference only suggests the possibility of fomite transmission based on (1) the presence of SARS-CoV-2 RNA on surfaces, and (2) experimental evidence of virus survival at specific conditions and high concentrations. Up to date, no study has "reported" fomite-mediated transmission. Replacing the word "reported" by "suggested" would be better.

**Authors' response: **Agreed. This was already commented on by Reviewer 1. We have revised as suggested.

**Peer reviewer's comment:**

**Discussion**

 In the following sentence, *"We identified one non-peer-reviewed (pre-print) systematic review that assessed fomite contamination in SARS-CoV-2."* perhaps what the authors intended to say was, "We identified one non-peer-reviewed (pre-print) systematic review that assessed SARS-CoV-2 contamination in fomites."?

**Authors' response: **We have made the correction. ****Now reads thus: “We identified one non-peer-reviewed (pre-print) systematic review that assessed SARS-CoV-2 contamination in fomites…”

**Peer reviewer's comment:**

**Figure 3**

 It would be more informative for the reader if the plot could distinguish between public spaces and rooms with COVID-19 patients. The authors could break the "other" category and include "isolation or quarantine room", since the most frequently contaminated spaces were quarantine rooms.

**Authors' response: **We understand the reviewer’s point here. However, we reported the settings are described by the study authors - only 2 studies were described as “hospital and quarantine”. Therefore we have not made any changes to the figure.

# Peer review: Reviewer response for version 1

**DOI:** 10.5256/f1000research.54776.r82053

**Published:** 2021-04-06

**Authors:** Emanuel Goldman

**Affiliations:** 1 Department of Microbiology, Biochemistry and Molecular Genetics, New Jersey Medical School, Newark, NJ, USA

In this manuscript, the authors have conducted an extensive comparison of many published studies attempting to assess the possibility of transmission of SARS-CoV-2 via fomites. Most of the studies reviewed involved samples from hospitals, although some studies were also from community settings. The authors report that none of the studies found infectious virus on fomites, and that viral RNA, while generally present to varying degrees, was likely of too poor quality in most instances to support infectivity anyway, based on relatively high cycle threshold (CT) values for the RNA samples.

 This is a useful snapshot of where we are in the pandemic, and why thinking in the field has increasingly moved towards the view that fomite transmission is not a significant source of infection. This reviewer believes the authors are too timid in drawing that conclusion, only stating in the Abstract *"Lack of positive viral cultures and variation in cycle thresholds create uncertainty about fomites as a mode of transmission."* I believe a more forceful statement about the insignificance of fomite transmission is warranted.

 In the second paragraph of the Introduction, the authors do not adequately characterize the content of some of the references cited.

 The authors write, *"it has been reported that SARS-CoV-2 can be transmitted indirectly through fomites or surfaces ^5^."* This is inaccurate; reference 5 is a review of work published up to that time, and at most, suggests the possibility that the virus can be transmitted through fomites. None of the papers reviewed in reference 5 showed indirect transmission through fomites. The authors must revise this sentence. My suggestion is to replace "reported" with "suggested", i.e., "it has been suggested that SARS-CoV-2 can be transmitted indirectly through fomites or surfaces ^5^."

 The opposite situation occurs in the very next sentence. The authors write, *"However, some authors have suggested that there is a low risk of transmission of SARS-CoV-2 through fomites ^6,7^." *Reference 6 is more than a suggestion; the authors report the absence of infectious virus on surfaces in hospitals treating COVID-19 patients. My suggestion here is the reverse of my previous one, in this case, replace "suggested" with "reported" for reference 6, i.e., "However, some authors have reported ^6^ or suggested ^7^ that there is a low risk of transmission of SARS-CoV-2 through fomites."

 In the last paragraph of the methods section, the authors write: *"One reviewer (IJO) assessed the risk of bias and extracted data from the included studies, and these were independently checked by a second reviewer (EAS)."* However, the reader is not given a clue as to what the authors mean by "bias" in this context. The absence of a definition and/or criteria for alleged "bias" makes this parameter meaningless in the manuscript. If the authors are to include this parameter, they must revise the manuscript to make clear what it is they are assessing as "bias", and how that assessment was made.

Are the rationale for, and objectives of, the Systematic Review clearly stated?

Yes

Is the statistical analysis and its interpretation appropriate?

Yes

Are sufficient details of the methods and analysis provided to allow replication by others?

Partly

Are the conclusions drawn adequately supported by the results presented in the review?

Yes

Reviewer Expertise:

Microbiology, Virology

I confirm that I have read this submission and believe that I have an appropriate level of expertise to confirm that it is of an acceptable scientific standard, however I have significant reservations, as outlined above.

# Response

**Published:** 2021-04-12

**Authors:** IGHO ONAKPOYA

**Affiliations:** University of Oxford, UK

We thank the reviewer for the useful feedback regarding our manuscript. In line with the reviewer's suggestions, we will be making the following revisions to the manuscript:

**ABSTRACT**

 We will revise the conclusions to reflect the results that the risk of transmission of SARS-CoV-2 through fomites is low.

**METHODS**

 In the second paragraph, we will replace "reported" with "suggested" (ref. 5) and replace "suggested" with "reported" (refs. 6&7).

 In the last paragraph of this section, we will expand the reporting of the methods used for risk of bias assessment - we will include the domains of bias assessed and how each domain was scored.

# Response

**Published:** 2021-05-14

**Authors:** IGHO ONAKPOYA

**Affiliations:** University of Oxford, UK

**Peer Reviewer's comment:**

 In this manuscript, the authors have conducted an extensive comparison of many published studies attempting to assess the possibility of transmission of SARS-CoV-2 via fomites. Most of the studies reviewed involved samples from hospitals, although some studies were also from community settings. The authors report that none of the studies found infectious virus on fomites, and that viral RNA, while generally present to varying degrees, was likely of too poor quality in most instances to support infectivity anyway, based on relatively high cycle threshold (CT) values for the RNA samples.

 This is a useful snapshot of where we are in the pandemic, and why thinking in the field has increasingly moved towards the view that fomite transmission is not a significant source of infection.

**Authors' response: **Thank you.

**Peer Reviewer's comment:**

 This reviewer believes the authors are too timid in drawing that conclusion, only stating in the Abstract *"Lack of positive viral cultures and variation in cycle thresholds create uncertainty about fomites as a mode of transmission."* I believe a more forceful statement about the insignificance of fomite transmission is warranted.

**Authors' response: **We agree with the reviewer and have revised the statement. Abstract conclusion now reads thus: “Lack of positive viral cultures suggests that the risk of transmission of SARS-CoV-2 through fomites is low.”

**Peer Reviewer's comment:**

 In the second paragraph of the Introduction, the authors do not adequately characterize the content of some of the references cited.

 The authors write, *"it has been reported that SARS-CoV-2 can be transmitted indirectly through fomites or surfaces ^5^."* This is inaccurate; reference 5 is a review of work published up to that time, and at most, suggests the possibility that the virus can be transmitted through fomites. None of the papers reviewed in reference 5 showed indirect transmission through fomites. The authors must revise this sentence. My suggestion is to replace "reported" with "suggested", i.e., "it has been suggested that SARS-CoV-2 can be transmitted indirectly through fomites or surfaces ^5^

**Authors' response: **We have amended the sentence. Now reads thus: “… it has been suggested that SARS-CoV-2 can be transmitted indirectly through fomites or surfaces…”

**Peer Reviewer's comment:**

 The opposite situation occurs in the very next sentence. The authors write, *"However, some authors have suggested that there is a low risk of transmission of SARS-CoV-2 through fomites ^6,7^." *Reference 6 is more than a suggestion; the authors report the absence of infectious virus on surfaces in hospitals treating COVID-19 patients. My suggestion here is the reverse of my previous one, in this case, replace "suggested" with "reported" for reference 6, i.e., "However, some authors have reported ^6^ or suggested ^7^ that there is a low risk of transmission of SARS-CoV-2 through fomites."

**Authors' response: **We have amended the sentence. Now reads thus: “… some authors have reported that there is a low risk of transmission of SARS-CoV-2 through fomites [6,7] and others have reported that the risk of such transmission is exaggerated [8]”.

**Peer Reviewer's comment:**

 In the last paragraph of the methods section, the authors write: *"One reviewer (IJO) assessed the risk of bias and extracted data from the included studies, and these were independently checked by a second reviewer (EAS)."* However, the reader is not given a clue as to what the authors mean by "bias" in this context. The absence of a definition and/or criteria for alleged "bias" makes this parameter meaningless in the manuscript. If the authors are to include this parameter, they must revise the manuscript to make clear what it is they are assessing as "bias", and how that assessment was made.

**Authors' response: **We have expanded the section on risk of bias assessment. Now reads thus: “We assessed the risk of bias using five domains from the QUADAS-2 criteria [9]; we adapted this tool because the included studies were not designed as diagnostic accuracy studies. The domains assessed were: (i) study description - was there sufficient description of methods to enable replication of the study? (ii) sample sources – was there a clear description of sample sources? (iii) description of results - was the reporting of study results and analysis appropriate? (iv) risk of bias - did the authors acknowledge any potential biases, if yes were any attempts made to address these biases? (v) applicability – is there any concern that the interpretation of test results differs from the study question? For each bias domain, the risk was judged as “low”, “unclear” or “high”.”
